# Antibiotic Resistance of Bacterial Isolates from Smallholder Poultry Droppings in the Guinea Savanna Zone of Nigeria

**DOI:** 10.3390/antibiotics11070973

**Published:** 2022-07-19

**Authors:** Oladeji Bamidele, Abdulmojeed Yakubu, Ehase Buba Joseph, Tunde Adegoke Amole

**Affiliations:** 1 International Livestock Research Institute (ILRI), P.M.B. 5320, Ibadan 200001, Nigeria; t.amole@cgiar.org; 2 Department of Biological Sciences, Kings University, Odeomu 220104, Nigeria; 3 Centre for Sustainable Agriculture and Rural Development, Department of Animal Science, Faculty of Agriculture, Shabu-Lafia Campus, Lafia, Nasarawa State University, Keffi 961101, Nigeria; abdulmojyak@gmail.com (A.Y.); ehasebubajoseph@gmail.com (E.B.J.)

**Keywords:** bacteria, antibiotics, multi-drug resistance, smallholder poultry, Nigeria

## Abstract

There is a growing risk of antibiotic resistance (AR) in smallholder poultry (SP). This study, therefore, aimed to investigate AR pattern of bacterial isolates from SP in the Guinea Savanna agro-ecological zone of Nigeria. A total of 120 fresh poultry droppings were aseptically collected, randomly, from two tropically adapted (FUNAAB Alpha and Noiler) and local chickens. The chickens were raised either using ethnoveterinary medicines (*n* = 60) or antibiotics (*n* = 60). Bacterial isolates were characterized and analyzed using standard protocols, and appropriate statistical tools. Compared to *Pseudomonas* spp. (2.5%) and *Klebsiella* spp. (5.8%), *Salmonella* spp. (57.5%) and *Escherichia coli* (34.2%) were the most prevalent (χ^2^ = 96.67; *p* < 0.001). Prevalence of bacterial species was significantly (*p* = 0.024; Odds Ratio = 2.552) influenced by antibiotics usage. All four species were multi-drug resistant. In total, 30% of the isolates had a multiple AR index ≥ 0.2. Bacterial isolates from FUNAAB Alpha (58.0%) and Noiler (44.0%) were highly resistant to quinolones, while isolates from the local chickens (22.6%) were most resistant to aminoglycosides. Bacterial species isolated from FUNAAB Alpha and local chickens exhibited the lowest and highest percentage of AR, respectively. Clustering of isolates with similar antibiogram revealed inter-species dependence with possibility for inter-species gene transfer. These findings provide a background to investigate the metagenomics of local and improved chickens for AR.

## 1. Introduction

Globally, poultry is a huge source of animal protein (eggs and meat), and in sub-Saharan Africa, poultry accounts for 24% of total meat production [1]. Smallholder farmers contribute significantly to the entire poultry value chain as over 80% of rural households practice smallholder poultry [2]. Specifically in Nigeria, 33% of the available total animal protein source comes from poultry production [1,3]. This has a significant implication on household food patterns, consumption of animal-sourced foods, food chain, and food safety.

Poultry production has been identified as a hot-spot for the development of antimicrobial resistance (AMR), and transfer of drug-resistant micro-organisms between food-producing animals and humans [4,5,6,7]. This is due to the high and chronic use of antibiotics, especially at sub-therapeutic levels, in commercial poultry. The indiscriminate use of antibiotics, both for therapeutic and non-therapeutic purposes (improved feeding, growth promoter), in poultry production systems presents a public health threat to humans [8,9,10,11]. This threat is heightened by the increased demand for animal protein owing to the growing population, and economic growth [11,12].

Poultry species have been intricately linked with zoonotic and foodborne diseases [13]. Approximately 91 million foodborne-related diseases resulting in 137,000 deaths per annum have been reported in Africa, making it one of the continents with the highest number of foodborne diseases [14]. This has led to mandated restrictions on the prophylactic use of antibiotics in chicken production in some countries [15,16,17]. Globally, the bacterial isolates from poultry droppings, that have been implicated in antibiotic resistance include *Escherichia coli* (*E. coli*), *Salmonella*, *Staphylococcus aureus*, *Campylobacter*, and *Proteus mirabilis* [17,18,19].

**Figure 1:**
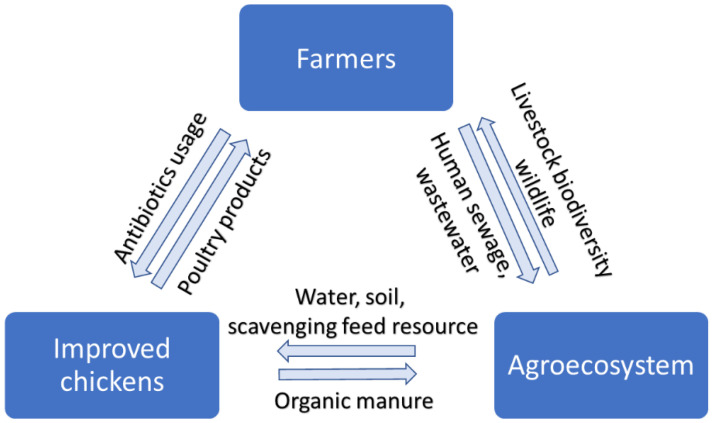
High-risk sources of antibiotic contamination and resistance in smallholder poultry production.

In recent times, due to increasing consumer preference for healthy foods, the demand for intensively raised poultry products is gradually being substituted with organically raised village chickens [20]. Village chickens are produced under scavenging, and semi-scavenging smallholder poultry production systems (SPPS) which have a lower risk of AMR [21]. The lower risk of AMR can be attributed to the unique characteristics of the SPPS which relies on ethnoveterinary practices for therapeutic and non-therapeutic animal care purposes [22,23]. However, the introduction of improved chicken genetics as a developmental program for improving SPPS [24,25] has contributed to a decline in farmers’ use of ethnoveterinary medicine and an increase in the indiscriminate use of antibiotics [26]. This intervention, though with positive developmental outcomes (food security, livelihoods) [27], may have unintended public health-related consequences for the environment, animals, and humans [11]. In addition, agroecological practices (organic manure, livestock biodiversity, scavenging feed resource base) [7,28,29], environmental elements (water, soil, wildlife, biocides) [30,31,32] as well as hygiene and sanitation levels (human sewage, wastewater, biosecurity) [33,34,35,36,37] within the SPPS, may act as reservoirs for the dissemination of AMR bacteria to the chickens which may eventually be transferred to humans through the food chain [38] (Figure 1). 

The production performance of improved chicken genetics for dual-purpose (meat, egg) functions within SPPS in Nigeria (FUNAAB Alpha, Noiler, ShikaBrown, Sasso, Kuroiler), and in some other African countries (Ethiopia, Tanzania) have been tested both under on-station and on-farm conditions [24,25,39,40]. Though the results show significant growth and laying performance compared to the local chickens, the potential of these breeds, relative to the local chickens, as a reservoir for AMR has not been investigated. Previous studies conducted in Nigeria have mainly reported antimicrobial resistance and bacteriological profiles of poultry droppings under intensive poultry production systems (commercial and institutional poultry farms) [41,42]. Therefore, the objective of this study was to differentially characterize the bacteriological profiles of poultry droppings in the local chickens and in two farmer-preferred, chicken breeds (FUNAAB Alpha and Noiler), and identify their antibiotic resistance patterns under SPPS in the Guinea Savanna agro-ecological zone of Nasarawa State, Nigeria.

## 2. Results

### 2.1. Microbial Count and Prevalence of Bacterial Pathogen

The microbial counts (cfu/mL) were not statistically significant (*p* > 0.05), and ranged from 4.64 to 5.19 × 10^5^ (Bacterial species), 4.60–4.81 × 10^5^ (Chicken genotype), 4.69–4.72 × 10^5^ (Sex), and 4.65–4.76 × 10^5^ (Antibiotics usage) (Table 1).

**Figure 2:**
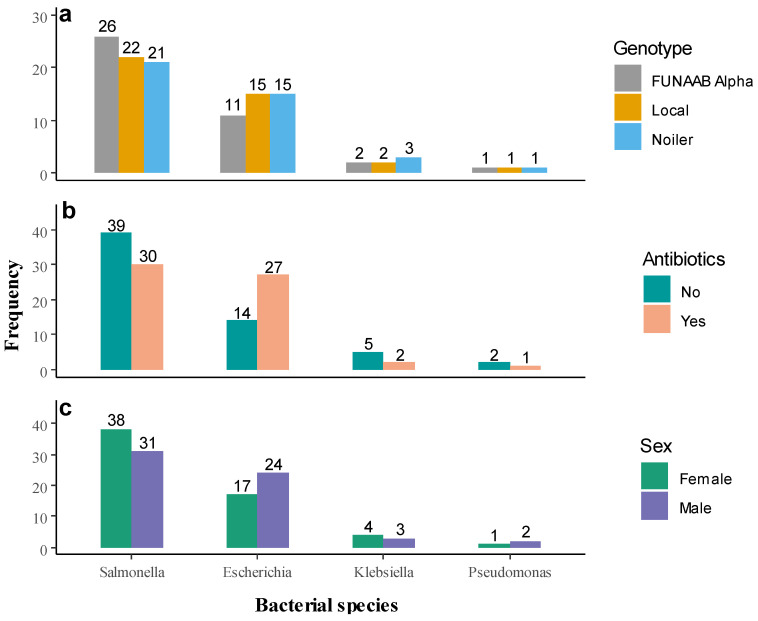
Prevalence of the bacterial species based on (**a**) genotypes of the chickens, (**b**) antibiotics usage, and (**c**) sex of the birds.

The numerical proportion of the bacterial species in the study area indicated that *Salmonella* spp. was statistically (χ^2^ = 96.67; *p* < 0.001) the most prevalent (69, 57.5%), followed by *E. coli* (41, 34.2%) and *Klebsiella* spp. (7, 5.8%). The least prominent was *Pseudomonas* spp. (3, 2.5%). However, there were no significant relationships between the prevalence of the bacterial species and chicken genotypes (FET = 2.252; *p* = 0.956), antibiotics usage (FET = 6.894; *p* = 0.060), and sex of birds (FET = 2.496; *p* = 0.508). Figure 2 shows the proportion of the bacterial species by genotypes, antibiotics usage, and sex.

In the binomial logistic regression model, only antibiotics usage of farmers was significantly (*p* = 0.024) important in predicting the occurrence of bacterial species (Table 2). There was a high risk of contracting bacterial species through antibiotics usage (Odds Ratio = 2.552; CI = 1.129–5.767). Among the isolates, the proportion of *E. coli* was noticeably higher in smallholder poultry farms where antibiotics were used (27, 65.9%) than in farms that did not use antibiotics (14, 34.1%). The Hosmer–Lemeshow test statistic indicated that the model fitted well (χ^2^ = 6.953; *p* = 0.542).

### 2.2. Antibiotic Resistance Pattern

The antibiotic resistance pattern shows that out of the 69 isolates for *Salmonella* spp., the percentage resistance to any of the antibiotics tested was highest with nalidixic acid (20.3%, *n* = 14), and lowest for ciprofloxacin (8.7%, *n* = 6). The resistance of *Pseudomonas* spp. (3 isolates) to both ciprofloxacin and sulfamethoxazole-trimethoprim(co-trimoxazole) was observed in only one of the isolates (33.3%, *n* = 1). *Klebsiella* spp. (7 isolates) had the highest percentage resistance to penicillin (42.9%, *n* = 3) compared to the other antibiotics. Among the antibiotic drugs, *E. coli* had the highest (19.5%, *n* = 8) and lowest (4.9%, *n* = 2) percentage resistance to perfloxacin and streptomycin, respectively. (Table 3). However, the effect of bacterial species on antibiotic drug resistance was not statistically significant (*p* > 0.05) for all drugs.

Across genotypes, antibiotic resistance of the bacterial isolates was only significantly (Kruskal–Wallis test value = 7.357; *p* = 0.025) different in local (17.5%) and Noiler (15.0%) birds for streptomycin. Within genotypes, bacterial isolates from FUNAAB Alpha exhibited the highest resistance to nalidixic acid (22.4%) while for Noiler, it was sulfamethoxazole-trimethoprim (20.0%). Bacterial species isolated from local chickens were more resistant to both sulfamethoxazole-trimethoprim (20.0%) and cephalexin (20.0%) (Table 4).

Isolates from male birds showed a higher resistance to penicillin (18.3%), unlike the isolates from female birds which exhibited more resistance to nalidixic acid (21.7%) (Table 5).

Compared to other antibiotic agents, penicillin (18.3%) had the highest percentage of antibiotic-free birds with bacterial isolates exhibiting antibiotic resistance while for the group given antibiotics, the percentage of birds with bacterial isolates showing antibiotic resistance was highest for nalidixic acid and sulfamethoxazole-trimethoprim (18.3%) (Table 6).

There was no significant (*p* > 0.05) effect of bacterial species resistance pattern based on class of antibiotic drugs (Table 7). Compared to other classes of antibiotics, *Salmonella* spp. (*n* = 37, 40.7%), *Klebsiella* spp. (*n* = 3, 33.3%), and *E. coli* spp. (*n* = 24, 47.1%) showed a higher percentage resistance to quinolones. In particular, *Salmonella* spp. (*n* = 13, 48.2%) and *E. coli* (*n* = 10, 37.0%) had a higher percentage resistance to second generation quinolones (Ciprofloxacin, ofloxacin)/fluroquinolones [43] than *Klebsiella* spp. (*n* = 3, 11.1%) and *Pseudomonas* spp. (*n* = 1, 3.7%). Chicken genotype significantly influenced (Kruskal–Wallis test value = 11.817; *p* = 0.003) the antimicrobial resistance of the bacterial isolates to the class of antibiotic drugs. While isolates from FUNAAB Alpha (*n* = 29, 58.0%) and Noiler (*n* = 22, 44.0%) were highly resistant to quinolones, isolates from local birds (*n* = 12, 22.6%) exhibited more resistance to aminoglycosides. The resistance pattern based on sex and antibiotics usage was the same for quinolones under both categories (38.4% and 46.3%), but these were not statistically significant (*p* > 0.05).

### 2.3. Multiple Antibiotic Resistance Index

**Figure 3:**
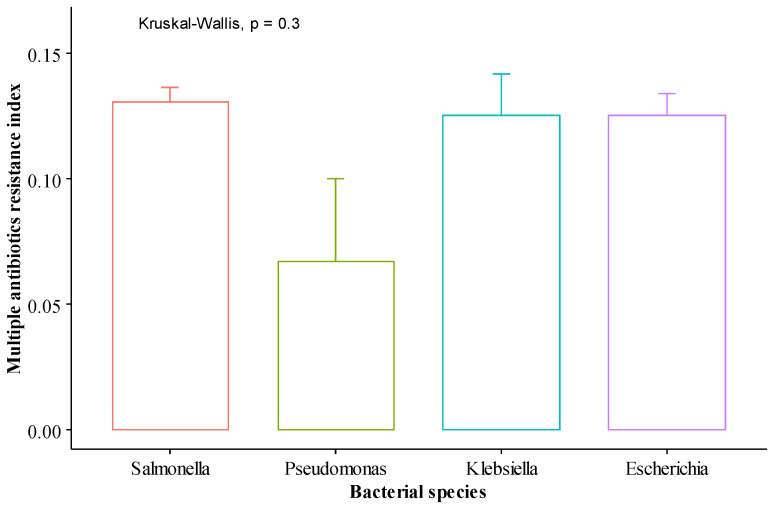
Multiple antibiotic resistance index of the bacterial species (*Salmonella* spp., *Pseudomonas* spp., *Klebsiella* spp., and *E. coli*). Bars on plots are standard errors.

**Figure 4:**
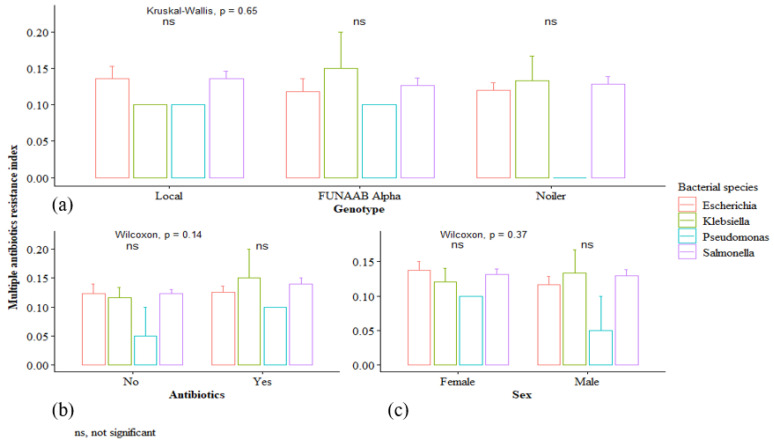
Effect of (**a**) genotype, (**b**) antibiotics usage, and (**c**) sex on multiple antibiotic resistance index of the bacterial species (*Salmonella* spp., *Pseudomonas* spp., *Klebsiella* spp., and *E. coli*). Bars on plots are standard errors.

Figure 3 shows that the multiple antibiotic resistance index (MAR) of the bacterial species was not significantly different (*p* > 0.05). Likewise, there were no significant differences (*p* > 0.05) in the MAR of the chickens either by genotype, antibiotics usage, or sex (Figure 4). The MAR indices were 0.2 (*Salmonella* spp. 22 (61.1%), *E. coli* 12 (33.3%), *Klebsiella* spp. 2 (5.6%)), 0.1 (*Salmonella* spp. 46 (57.6%), *E. coli* 27 (33.7%) *Klebsiella* spp. 5 (6.2%), *Pseudomonas* spp. 2 (2.5%)), and 0.0 (*Salmonella* spp. 1 (25.0%), *E. coli* 2 (50.0%), *Pseudomonas* spp. 1 (25.0%)) across the bacterial species, respectively. The percentage proportion of the bacterial species isolated from FUNAAB Alpha (0.2: 12 (33.3%); 0.1: 26 (32.5%, and 0.0: 2 (50.0%)), local (0.2: 14 (38.9%), 0.1: 25 (31.2%), and 0.0: 1 (25.0%)) and Noiler (0.2: 10 (27.8%), 0.1: 29 (36.3%), and 0.0: 1 (25.0%)) chickens for the MAR indices (0.2, 0.1, and 0.0) varied, respectively. The distribution of the MAR index (0.2, 0.1, 0.0) in chickens not given antibiotics was 14 (38.9%), 44 (55.0%), and 2 (50.0%) while for chickens treated with antibiotics, it was 22 (61.1%), 36 (45.0%), and 2 (50.0%), for the respective indices.

### 2.4. Hierarchical Clustering of Bacterial Isolates

**Figure 5:**
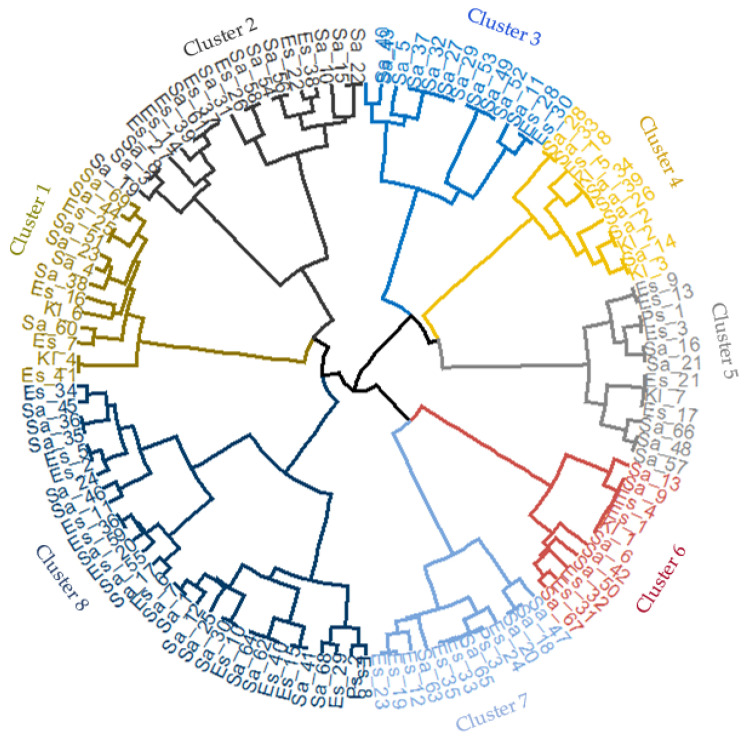
A cluster dendrogram of bacterial species based on the antibiotic resistance patterns, antibiotics usage, and genotypes. Sa: *Salmonella* spp., Es: *E. coli*, Kl: *Klebsiella* spp., Ps: *Pseudomonas* spp.

The hierarchical clustering of the bacterial isolates (120), with respect to their antibiogram across the 10 antibiotic drugs, revealed an eight-cluster dendrogram (Figure 5). The dendrogram showed similarities in the antimicrobial susceptibility profiles of the four bacterial species. The composition of the clusters was as follows: cluster 1, 13 isolates (7 *Salmonella* spp., 4 *E. coli*, 2 *Klebsiella* spp.); cluster 2, 18 isolates (11 *Salmonella* spp., 7 *E. coli*), cluster 3, 13 isolates (11 *Salmonella* spp., 2 *E. coli*); cluster 4, 11 isolates (7 *Salmonella* spp., 1 *E. coli*, 3 *Klebsiella* spp.); cluster 5, 12 isolates (5 each of *Salmonella* spp. and *E. coli*, 1 each of *Pseudomonas* spp. and *Klebsiella* spp.); cluster 6, 11 isolates (6 *Salmonella* spp, 4 *E. coli*, 1 *Klebsiella* spp.); cluster 7, 12 isolates (5 *Salmonella* spp., 6 *E. coli* and 1 *Pseudomonas* spp.), and cluster 8 which was the largest group with 30 bacterial isolates comprised *Salmonella* spp. (17), *E. coli* (12), and *Pseudomonas* spp. (1).

A high percentage (53.3–90.9%) of bacterial species isolated from antibiotic-free chickens was found in clusters 3, 4, 6, and 8 as against the predomination of isolates from antibiotic-treated chickens in clusters 1 (53.8%), 2 (61.1%), and 7 (75.0%) (Table 8). Compared to the local chickens which was the predominant genotype in clusters 2 and 6, Noiler and FUNAAB Alpha were mostly grouped into clusters 3, 4, and 5, and clusters 1, 7, and 12, respectively.

Across the clusters, the bacterial species presented a higher susceptibility to cephalexin (93.7%), gentamycin (93.1%), and perfloxacin (90.7%) than nalixidic acid (89.7%), amoxycillin-clavulanic acid (88.6%), streptomycin (88.5%), ofloxacin (87.5%), ciprofloxacin (85.8%), penicillin (83.9%), and sulfamethoxazole-trimethoprim (78.9%). On average, cluster 6 (96.4%) showed the highest susceptibility to all antibiotic drugs followed by clusters 5 (90.0%), 8 (88.3%), 4 (87.3%), 2 (87.2%), 7 (86.7%), 1 (85.4%), and 3 (84.6%) in that order. Cephalosporins were largely effective (100%) against all bacterial isolates within the clusters, except in clusters 6 (90.9%) and 7 (75%). Quinolones (cluster 5), sulfonamides (clusters 2, 7, and 8), β-lactams (clusters 3–6), and penicillins (clusters 1, 5–8)/aminoglycosides (clusters 1, 4–7) were only largely effective in 12.5%, 37.5%, 50%, and 62.5% of the clusters, respectively.

## 3. Discussion

The indiscriminate use of antimicrobial agents particularly in poultry has led to the development of antimicrobial resistance in food-borne pathogens. Therefore, the spread of antibiotic-resistant bacteria from food animals to humans and the environment is a major global health concern. Four bacterial species were identified in the present study through morphological and biochemical characteristics. Compared to the other bacterial species, the higher prevalence of *Salmonella* spp. (57.5%) and *E. coli* (34.2) observed in this study could be an indication of their superior adaptability and fitness within the harsh environment of the smallholder poultry production systems. The prevalent rates are comparable to the values previously reported for *E. coli* (39.0%) [18] and *Salmonella* spp. (59.5%) [44]. However, higher *E. coli* (59.2%) and lower *Salmonella* spp. (15.5%) prevalence rates have also been reported [45]. Overall, our result is comparable with previous findings on the incidence and prevalence of *Salmonella* spp. and *E. coli* in poultry which therefore affirms the endemic nature of these bacterial species in poultry [46]. The endemic nature of these species may well be attributed to the inadequate measures aimed at preventing and controlling the spread of infections, as well as poor hygiene, husbandry, and biosecurity practices on the part of the farmers. According to WHO [47], non-availability of clean water sources, poor sanitation, and inadequate infection prevention and control exacerbate microbial spread, some of which could resist antimicrobial treatments. 

The results of the binary regression indicated that antibiotics usage significantly influenced the occurrence of bacterial species. Contrary to expectation, there was a pronounced occurrence of *E. coli* in farms where antibiotics were used. This is because antibiotics are known to inhibit bacterial growth [6,48]. This phenomenon could be due to a lower concentration of antibiotics used by the farmers, the potential antibiotic resistance capability of *E. coli*, and other possible management (feed and water troughs) and environmental sources (soil, water, scavenging feed resource) of bacterial contamination. Soil, water, and air have been reported as high-risk sources of *E. coli* contamination in poultry farms, and these sources may be affected by location, season, and agroecology [49,50,51]. 

Medium to low antibiotic resistance was observed in the present study. This could be due to the classes and types of the drugs tested based on availability at the time of the analysis. Low resistance of *E. coli* to gentamicin (6.0%), ciprofloxacin (10.0%), and ofloxacin (10.0%) had been reported earlier [52]. There is the tendency that the pattern of *Salmonella* spp., *E. coli*, *Klebsiella* spp., and *Pseudomonas* spp. resistance to antibiotics may change if other conventional veterinary drugs are tested. This is because high resistance to antibiotics such as tetracycline (89.4%), cloxacillin (100.0%), and erythromycin (100.0%) [53] in Nigeria, amoxycillin (88.4%) in Uganda [46], and ampicillin (91.7%) in Ethiopia [18] has been reported in poultry. These observations are consistent with the findings of Nhung [54] in a comprehensive review where the antibiotic resistance of *E. coli* and *Salmonella* spp., among others, were found to vary with the classes and types of antibiotic drugs used. 

Bacterial species isolated from FUNAAB Alpha (44.6%) and Noiler (33.8%) chickens were more highly resistant to quinolones compared to the local birds (21.6%). This may have resulted from the indiscriminate use of quinolone-based antibiotics by farmers in the study. Compared to the other two genotypes, our results suggest that drugs classified as aminoglycosides and cephalosporins might have been abused by farmers during the production of the local chickens within our study area. This is because the local birds account for over half of the bacterial isolates with antibiotic resistance to those two drug classes. The high resistance of the bacterial isolates, from the local birds, to aminoglycosides (57.2%) and cephalosporins (57.1%) may be due to the misuse of antibiotics either directly within the production systems or indirectly from the environment. Potentially, this increases the risk of exposure to antimicrobial resistance by humans from smallholder poultry [55,56]. Bacterial isolates from FUNAAB Alpha chickens had a lower (20%) percentage of antibiotic resistance to both penicillins and sulphonamides, compared to Noiler (40%) and the local (40%) chickens. The effect of the three genotypes (Noiler, FUNAAB Alpha, and local) on the antimicrobial resistance of the bacterial isolates, across the various drug classes was significantly different (*p* < 0.05). Overall, ranking of the genotypes based on the antibiotic resistance profile of the bacterial isolates shows FUNAAB Alpha and the local chickens as having the lowest and highest risk of antimicrobial resistance, respectively. 

Our study showed the presence of multi-drug resistance with a MAR index ranging from 0.0 to 0.2. However, this range is lower than that previously reported (0.5–0.9) for exotic poultry birds raised under intensive (commercial) production systems in Nigeria [57]. This difference in the range of MAR index between the intensively raised poultry, and smallholder poultry could be as a result of an extremely high dependency and indiscriminate use of antibiotics on commercial farms. This suggests that smallholder poultry presents a lower risk of antibiotic resistance to humans and the environment. 

Although there were no significant differences in the MAR index of the isolates from the three genotypes, local chickens were observed to have a higher percentage (38.9%) of birds with MAR index ≥ 0.2, compared to FUNAAB Alpha (33.3%) and Noiler (27.8%). This may have been due to the good scavenging ability of the birds within a production environment contaminated with antibiotics, either directly (feed, water) or indirectly (human sewage, wastewater) [57,58,59]. The scavenging behavior is an adaptive trait of local chickens influenced by several genetic factors, and contributes to their survivability and resilience, compared to the exotic and improved breeds [60]. As expected, chickens not treated with antibiotics had a lower percentage (38.9%) of isolates with MAR index ≥ 0.2, compared to those treated with antibiotics (61.1%). Still, the presence of isolates with MAR ≥ 0.2 in chickens not given antibiotics suggests high-risk sources of antibiotic contamination within the farmers’ production environment. 

Less than one-third (30%) of the isolates had MAR index ≥ 0.2. *Salmonella* spp. had the highest percentage (61.1%) of isolates with MAR index ≥ 0.2 followed by *E. coli* (33.3%) and *Klebsiella* spp. (5.6%). According to Afunwa et al. [57] and Raiz et al. [61], organisms with MAR index ≥ 0.2 potentially possess antibiotic resistance genes in their plasmids. All three species have been listed on the global priority list of antibiotic-resistant bacteria [62]. The presence of fluroquinolone-resistant *Salmonella* spp., and cephalosporin-resistant *E. coli* in the droppings of chickens in the study area, were of particular concern because both have been grouped as high and critical priority pathogens for antibiotic resistance in humans, respectively [62]. In addition, our study shows that these three bacterial species had a high isolate–drug combination for ciprofloxacin, ofloxacin, nalidixic acid, and perfloxacin, thereby suggesting an abuse of quinolone-based antibiotics within the study area. Additionally, the bacterial isolates showed a moderately high (Noiler: 42%; Local: 45%; FUNAAB Alpha: 54%) percentage of resistance for fluroquinolones, β-lactam, cephalosporins, and penicillin classes of antibiotics across the three chicken genotypes. This increases the potential of the chickens to serve as reservoirs for antimicrobial resistance because these drug classes are essential, first or second choice empirical treatment options for infectious diseases in humans [63]. According to Murray et al. [64], resistance to second generation quinolones (Fluroquinolones) and beta-lactam ringed antibiotics were implicated in over 70% of deaths attributed to antimicrobial resistance in humans across all pathogens. In this study, *Salmonella* spp. (52.8%) and *E. coli* (34.7%) accounted for a large majority (87.5%) of the bacterial isolates exhibiting multi-antibiotic, drug class resistance in the chickens.

The pattern of clustering observed for the bacterial species suggests a co-existence of *Salmonella* spp., *E. coli*, *Klebsiella* spp., and *Pseudomonas* spp. under similar management conditions which could influence horizontal gene transfer between the species. The implication of this is that diagnostics that depend on species-specific bacterial targets may be impeded, resulting in treatment failure. Additionally, such inter-species dependence might contribute to the survivability and resistance of bacterial species within an agroecosystem. This could constitute environmental hazards, thereby posing a greater risk to animal and human health [65,66]. Our findings are congruous to the submission of Hull et al. [67] where high-level inter-species gene transfer was observed between *C. coli* and *C. jejuni* in chickens. The results are also supported by a recent study on the use of hierarchical clustering in identifying similarities in pathogen–drug combinations for *E. coli* isolates of poultry and other mammals [68].

Susceptibility of the bacterial species, between and within the identified clusters, varied across the antibiotic drug classifications. Cephalosporins were completely effective against all bacterial species in three-quarters (75%) of the clusters. Sulfonamides and cephalosporins had the lowest (79.3%) and highest (95.7%) susceptibility rates, respectively. The high resistance to sulfonamides may be due to the misuse and overuse of this group of antibiotics by farmers. Several studies have reported the predominant use of co-trimoxazole (sulfamethoxazole-trimethoprim) and sulfaquinoxaline in poultry farms in Nigeria [26,41,53,69]. According to Bamidele et al. [26], sulfonamides are ranked third on the list of antibiotics commonly used in smallholder poultry production systems in Nigeria. 

## 4. Materials and Methods

### 4.1. Sampling Location and Farmer Selection

Samples of fresh poultry droppings belonging to three chicken genotypes (FUNAAB Alpha, Noiler, Local) were collected from smallholder poultry households located in two villages (Gitta Mbasha and Karshi) of Nasarawa State. The geographical location and agro-ecological features of Nasarawa State have been described by Yakubu et al. [24] and Bamidele and Amole [70]. The two villages were selected based on the practice of ethnoveterinary medicine (Gitta Mbasha) and administration of synthetic (pharmaceuticals) antibiotics (Karshi) to the flock by the farmers. In Gitta Mbasha, the farmers used leaves, bulbs, barks, or seeds of a wide range of plants as ethnoveterinary medicines. Some of these plants include garlic (*Allium sativa*), onion (*Allium cepa*), neem tree (*Azadirachta indica*), and velvet bean (*Mucuna pruriens*). The antimicrobial drugs administered by the farmers in Karshi village were Keproceryl^®^ (a mix of oxytetracycline, erythromycin, colistin and streptomycin), Amprocox^®^ (Amprolium + Sulphaquinoxaline), Septrin^®^ (Sulfamethoxazole-Trimethoprim), ampicillin and Ampiclox^®^ (Ampicillin + Cloxacillin). A total of 18 farmers (9 per village) were randomly selected from a pool of 50 farmers (25 per village) who had previously received 10 pre-vaccinated chickens at 5 weeks old, of either FUNAAB Alpha or Noiler breed. The improved chicken breeds (FUNAAB Alpha and Noiler), and the local birds (of similar age group) already in the households, were tagged (wing) and raised together by the farmers under the same management conditions (semi-scavenging) for 16 weeks (July–November 2021). Field officers (2) were recruited and attached to the villages to monitor the performance of the birds throughout the study period. 

### 4.2. Collection of Samples

Fecal samples were collected from the chickens (*n* = 20) of each of the three genotypes, for each enrolled village, at 21 weeks of age. A total of 120 fresh poultry droppings were aseptically collected using sterile spatulas from randomly selected apparently healthy chickens (10 cocks and 10 hens) of three genotypes (20 birds per genotype). The genotypes were the two tropically adapted breeds (FUNAAB Alpha, Noiler), and the local (indigenous) chickens. The samples (1 g each), which were placed into sterile universal sampling bottles, were kept in a mobile box containing ice packs and transported immediately to the laboratory for microbiological analyses. 

### 4.3. Isolation and Identification of Bacteria Isolates

Bacteriological examinations were carried out in the laboratory using standard procedures for aerobic bacteria. For the detection of *Salmonella* spp., a representative portion of the fecal sample was inoculated into Selenite F Broth to prevent the growth of other bacterial species apart from Salmonella and Shigella. Then, a loopful of enriched sample was streaked on *Salmonella-Shigella* (SS) agar and incubated at optimum temperature of 36–37 °C for 24 h. The presumptive identification of the particular monotype (*Salmonella* spp.) was subjected to morphological and biochemical characteristics such as shape, size, surface texture, edge, elevation and color, motility, Gram staining, and biochemicals (indole production, urease, oxidase, catalase, lactase, and citrate) [71,72]. In order to identify *E. coli*, *Pseudomonas* spp., and *Klebsiella* spp., the samples were grown on MacConkey agar (for mixed growth) and incubated at 36–37 °C for 24 h. Then, each single colony was sub-cultured to obtain a pure culture. The plates were then examined for growth. Morphological and biochemical characteristics of the bacteria colony were used for confirmation. Microbial counts of *Salmonella* spp., *E. coli*, *Pseudomonas* spp., and *Klebsiella* spp. were conducted using the pour plate technique. In total, 0.1 mL of serially diluted suspensions (10^−2^, 10^−4^, 10^−6^, and 10^−8^) was mixed in cooled molten agar medium and poured into a petri dish. The plate was then rotated for proper mixing. It was allowed to set and then incubated at 37 °C for 2 days. Colonies that appeared throughout the medium were counted and multiplied by the dilution factor to obtain the number of bacteria in the original suspension as follows [72]:

$$\mathrm{Colony}\;\mathrm{forming}\;\mathrm{units}\;\mathrm{per}\;\mathrm{ml}\;\left(\mathrm{cfu}/\mathrm{mL}\right)=\mathrm{Colonies}\;\left(\mathrm{average}\right)\times \frac{\mathrm{Dilution}\;\mathrm{factor}}{\mathrm{Volume}\;\mathrm{plated}}$$

Selenite F Broth and SS agar were produced by TM Media (Titan Biotech. Ltd., Rajasthan, India) while MacConkey agar was produced by HIMEDIA (HiMedia Laboratories Pvt. Ltd., Mumbai, India). The biochemicals were purchased from L:S-BIOTECH (LIFESAVE BIOTECH, San Diego, CA, USA). The agar media and biochemicals were prepared in the laboratory according to the manufacturers’ instructions.

### 4.4. Antimicrobial Susceptibility Testing

The antibiotic susceptibility test of bacterial pathogen isolates was determined by the disk diffusion method and interpreted as described by Cheesbrough [71] and Ochei and Kolhatkar [72]. The plates were incubated aerobically at an optimum temperature of 36–37 °C for 24 h. The bacterial species were tested against 10 antibiotics (with their respective concentrations) belonging to six different classes, namely, Quinolones (Ciprofloxacin (10 μg), Ofloxacin (10 μg), Nalidixic acid (30 μg), Perfloxacin (10 μg)), β-lactams (Augmentin: amoxicillin and clavulanic (30 μg)), Aminoglycosides (Gentamicin (10 μg), and Streptomycin (30 μg)), Penicillins (Penicillin (10 μg)), Sulfonamides (Co-trimoxazole: sulfamethoxazole-trimethoprim (10 μg)), and Cephalosporins (Ceporex: Cephalexin (30 μg)) as described by WHO [73]. The antibiotics were manufactured by TM Media (Titan Biotech Ltd., Delhi, India). The multiple antibiotic resistance (MAR) index for each isolate was manually calculated as described by Krumperman [74]. 

### 4.5. Statistical Analysis

The microbial counts were subjected to ANOVA and T-test based on the bacterial species (*Salmonella* spp., *Pseudomonas* spp., *Klebsiella* spp., and *E. coli*), genotypes (Local, FUNAAB Alpha, and Noiler), sex of birds (male and female), and antibiotic usage by farmers (Yes: use of pharmaceuticals/synthetic antibiotics, and No: practice of ethnoveterinary medicine). The proportion of the prevalence of the bacterial species was determined using Chi-square (χ^2^) goodness-of-fit test. Then, Fisher’s exact test (FET) was used to evaluate whether there were significant differences between the prevalence of the bacterial species and the genotypes, sex, and antibiotics usage. A binomial logistic regression model was applied to determine the effects of independent factors (genotype, sex, and antibiotics usage) on the odds of occurrence of bacterial species (*Salmonella* spp. and *E. coli* only) at 95% confidence level. *Pseudomonas* spp. and *Klebsiella* spp. were excluded from the logistic regression due to their relatively low numbers. The model fit was examined using the Hosmer–Lemeshow test (a non-significant χ^2^ value (*p* > 0.05) indicates a good fit of the model) which explores whether the predicted and observed probabilities were the same [75]. The non-parametric Kruskal–Wallis H test was used to compare the antibiotic resistance rates of the different bacterial species relative to the genotype, sex, and antibiotics usage.

Significant differences were declared at α = 0.05. Prior to analysis, Levene’s test of homogeneity of variance and Shapiro–Wilk test of normality were conducted on the data to validate the parameter assumptions. The descriptive and inferential statistical analyses were conducted using IBM-SPSS [76] and R (version 4.1.2) [77].

In order to identify clusters of bacterial isolates with a similar antibiogram (a profile of how susceptible or resistant the bacterial isolates are to a variety of antibiotics), resistance and susceptibility were coded as 0 and 1, respectively. Hierarchical clustering was conducted using Euclidean distance and Ward’s method of distance measures. Ward’s method (Ward.D) was selected after comparing with other distance measures (complete, average linkage, single, ward.D2). In addition to visualizing the dendrogram, the elbow method was used to determine the optimal number of clustering. The cluster analysis was conducted in R (version 4.1.2) using the package factoextra (version 1.0.7) [78].

## 5. Conclusions

This is the first report on antibiotic resistance patterns of bacterial species in the improved, tropically adapted birds under scavenging and semi-scavenging production systems in Nigeria. *Salmonella* spp. and *E. coli* were the most prevalent bacteria in the study area. The use of antibiotics in smallholder poultry farms influenced the prevalence of bacterial species. Multidrug resistance was observed in the four bacterial species. Among the genotypes, isolates from FUNAAB Alpha exhibited the lowest percentage of antibiotic resistance while isolates from the local chickens had the highest percentage. Bacterial species isolated from FUNAAB Alpha and Noiler chickens were more resistant to quinolones than the local birds, although isolates from the local chickens showed more resistance to aminoglycosides. This study provided insights into the possible co-existence of *Salmonella* spp., *E. coli*, *Klebsiella* spp., and *Pseudomonas* spp. within the agroecosystem which could potentially influence horizontal gene transfer between the species, thereby increasing the threat of antibiotic resistance to animals, humans, and the environment. However, further research is required to differentially analyze the metagenomic profiles of the tropically adapted breeds relative to the local birds for the presence of antibiotic-resistant genes which may be associated with antimicrobial resistance in chickens and humans. 

## Figures and Tables

**Table 1:** The microbial counts (cfu/mL) of bacterial isolates from poultry droppings.

Variables	Number	Mean ± S.E. (×10^5^)	Minimum	Maximum
Bacterial species				
*Salmonella* spp.	69	4.64 ± 0.20	2.0	7.4
*Pseudomonas* spp.	3	5.10 ± 0.47	4.4	6.0
*Klebsiella* spp.	7	5.19 ± 0.55	3.9	7.6
*E. coli*	41	4.70 ± 0.19	2.1	7.2
Chicken genotype				
Local	40	4.81 ± 0.26	2.0	7.6
FUNAAB Alpha	40	4.71 ± 0.22	2.3	7.3
Noiler	40	4.60 ± 0.23	2.1	7.4
Sex				
Male	60	4.69 ± 0.18	2.1	7.4
Female	60	4.72 ± 0.20	2.0	7.6
Antibiotics usage				
No	60	4.76 ± 0.22	2.0	7.6
Yes	60	4.65 ± 0.17	2.1	7.4

S.E. = standard error, means along column for each group were not significantly different (*p* > 0.05).

**Table 2:** Risk factors associated with the occurrence of bacterial isolates in chicken.

Parameters	β	S.E.	Wald	df	*p*-Value	Odds Ratio	95% C.I.
Chicken genotype (ref: Local)			1.276	2	0.528		
FUNAAB Alpha	−0.442	0.507	0.760	1	0.383	0.643	0.238 − 1.736
Noiler	0.106	0.493	0.046	1	0.830	1.112	0.423 − 2.921
Sex (ref: male)							
Female	−0.541	0.412	1.731	1	0.188	0.582	0.260 − 1.304
Antibiotics usage (ref: No)							
Yes	0.937	0.416	5.071	1	0.024 *	2.552	1.129 − 5.767
Constant	−0.667	0.456	2.138	1	0.144	0.513	

β = regression coefficient; S.E. = standard error; df = degree of freedom; C.I. = confidence interval; ref = reference category; * significant at *p* < 0.05.

**Table 3:** Antibiotic resistance pattern of the bacterial species.

Antibiotic Agents	Bacterial Species/Number (*n*) of Isolates				Total *n* = 120	Kruskal–Wallis Test	*p*-Value
	*Salmonella* spp. *n* = 69	*Pseudomonas* spp. *n*= 3	*Klebsiella* spp. *n* = 7	*E. coli* *n* = 41			
Ciprofloxacin							
Susceptibility	63 (91.3)	2 (66.7)	6 (85.7)	35 (85.4)	106 (88.3)	2.335	0.506
Resistance	6 (8.7)	1 (33.3)	1 (14.3)	6 (14.6)	14 (11.7)		
Ofloxacin							
Susceptibility	62 (89.9)	3 (100.0)	5 (71.4)	37 (90.2)	107 (89.2)	2.705	0.439
Resistance	7 (10.1)	0 (0.0)	2 (28.6)	4 (9.8)	13 (10.8)		
Nalidixic acid							
Susceptibility	55 (79.7)	3 (100.0)	7 (100.0)	35 (85.4)	100 (83.3)	2.751	0.432
Resistance	14 (20.3)	0 (0.0)	0 (0.0)	6 (14.6)	20 (16.7)		
Perfloxacin							
Susceptibility	59 (85.5)	3 (100.0)	7 (100.0)	33 (80.5)	102 (85.0)	2.413	0.491
Resistance	10 (14.5)	0 (0.0)	0 (0.0)	8 (19.5)	18 (15.0)		
Gentamicin							
Susceptibility	62 (89.9)	3 (100.0)	7 (100.0)	38 (92.7)	110 (91.7)	1.250	0.741
Resistance	7 (10.1)	0 (0.0)	0 (0.0)	3 (7.3)	10 (8.3)		
Amoxycillin-Clavulanic acid							
Susceptibility	61 (88.4)	3 (100.0)	6 (85.7)	34 (82.9)	104 (86.7)	1.134	0.769
Resistance	8 (11.6)	0 (0.0)	1 (14.3)	7 (17.1)	16 (13.3)		
Sulfamethoxazole-Trimethoprim							
Susceptibility	58 (84.1)	2 (66.7)	6 (85.7)	34 (82.9)	100 (83.3)	0.654	0.884
Resistance	11 (15.9)	1 (33.3)	1 (14.3)	7 (17.1)	20 (16.7)		
Streptomycin							
Susceptibility	58 (84.1)	3 (100.0)	7 (100.0)	39 (95.1)	107 (89.2)	4.546	0.208
Resistance	11 (15.9)	0 (0.0)	0 (0.0)	2 (4.9)	13 (10.8)		
Penicillin							
Susceptibility	61 (88.4)	3 (100.0)	4 (57.1)	37 (90.2)	105 (87.5)	6.605	0.086
Resistance	8 (11.6)	0 (0.0)	3 (42.9)	4 (9.8)	15 (12.5)		
Cephalexin							
Susceptibility	60 (87.0)	3 (100.0)	6 (85.7)	37 (90.2)	106 (88.3)	0.709	0.871
Resistance	9 (13.0)	0 (0.0)	1 (14.3)	4 (9.8)	14 (11.7)		

Numbers in parentheses are percentages.

**Table 4:** Antibiotic resistance (number, %) pattern based on chicken genotype.

Antibiotic Agents	Chicken Genotype			Kruskal–Wallis Test	*p*-Value
	Local *n* = 40	FUNAAB Alpha *n* = 40	Noiler *n* = 40		
Ciprofloxacin	2 (5.0)	7 (17.5)	5 (12.5)	3.047	0.218
Ofloxacin	3 (7.5)	6 (15.0)	4 (10.0)	1.198	0.549
Nalidixic acid	4 (10.0)	9 (22.4)	7 (17.5)	2.261	0.323
Perfloxacin	5 (12.5)	7 (17.5)	6 (15.0)	0.389	0.823
Gentamicin	5 (12.5)	3 (7.5)	2 (5.0)	1.515	0.469
Amoxycillin-Clavulanic acid	5 (12.5)	7 (17.5)	4 (10.0)	1.001	0.606
Sulfamethoxazole-Trimethoprim	8 (20.0)	4 (10.0)	8 (20.0)	1.904	0.386
Streptomycin	7 (17.5)	0 (0.0)	6 (15.0)	7.357	0.025 *
Penicillin	6 (15.0)	3 (7.5)	6 (15.0)	1.360	0.507
Ceporex	8 (20.0)	4 (10.0)	2 (5.0)	4.491	4.491

* Significant at *p* < 0.05.

**Table 5:** Antibiotic resistance (number, %) pattern based on sex of birds.

Antibiotic Agents	Sex		Kruskal–Wallis Test	*p*-Value
	Male *n* = 60	Female *n* = 60		
Ciprofloxacin	7 (11.7)	7 (11.7)	0.000	1.000
Ofloxacin	4 (6.7)	9 (15.0)	2.502	0.114
Nalidixic acid	7 (11.7)	13 (21.7)	2.180	0.140
Perfloxacin	10 (16.7)	8 (13.3)	0.263	0.608
Gentamicin	6 (10.0)	4 (6.7)	0.436	0.509
Amoxycillin-Clavulanic Acid	7 (11.7)	9 (15.0)	0.688	0.407
Sulfamethoxazole-Trimethoprim	10 (16.7)	10 (16.7)	0.263	0.608
Streptomycin	4 (6.7)	9 (15.0)	2.161	0.142
Penicillin	11 (18.3)	4 (6.7)	3.337	0.068
Cephalexin	7 (11.7)	7 (11.7)	0.086	0.769

**Table 6:** Antibiotic resistance (number, %) pattern based on antibiotics usage of farmers.

Antibiotic Agents	Antibiotics Usage			
	No *n* = 60	Yes *n* = 60	Kruskal–Wallis Test	*p*-Value
Ciprofloxacin	4 (6.7)	10 (16.7)	1.179	0.278
Ofloxacin	6 (10.0)	7 (11.7)	0.036	0.849
Nalidixic acid	9 (15.0)	11 (18.3)	0.098	0.754
Perfloxacin	9 (15.0)	9 (15.0)	0.028	0.867
Gentamicin	4 (6.7)	6 (10.0)	0.292	0.589
Amoxycillin-Clavulanic acid	7 (11.7)	9 (15.0)	0.461	0.497
Sulfamethoxazole-Trimethoprim	9 (15.0)	11 (18.3)	0.119	0.730
Streptomycin	6 (10.0)	7 (11.7)	0.024	0.877
Penicillin	11 (18.3)	4 (6.7)	1.827	0.177
Cephalexin	8 (13.3)	6 (10.0)	0.188	0.665

**Table 7:** Antimicrobial resistance (number, %) patterns based on class of antibiotic drugs.

Parameters	Factors				Kruskal–Wallis Test	*p*-Value
	Bacterial Species					
Antibiotic Class	*Salmonella* spp. *n* = 91	*Pseudomonas* spp. *n* = 2	*Klebsiella* spp. *n* = 9	*E. coli* *n* = 51		
Quinolones	37 (40.7)	1 (50.0)	3 (33.3)	24 (47.1)	0.886	0.829
β-lactams	8 (8.8)	0 (0.0)	1 (11.1)	7 (13.7)		
Penicillins	8 (8.8)	0 (0.0)	3 (33.3)	4 (7.8)		
Aminoglycosides	18 (19.8)	0 (0.0)	0 (0.0)	5 (9.8)		
Sulfonamides	11 (12.1)	1 (50.0)	1 (11.1)	7 (13.7)		
Cephalosporins	9 (9.9)	0 (0.0)	1(11.1)	4 (7.8)		
	Chicken genotype					
Antibiotic Class	Local *n* = 53	FUNAAB Alpha *n* = 50	Noiler *n* = 50			
Quinolones	14 (26.4)	29 (58.0)	22 (44.0)		11.817	0.003
β-lactams	5 (9.4)	7 (14.0)	4 (8.0)			
Penicillins	6 (11.3)	3 (6.0)	6 (12.0)			
Aminoglycosides	12 (22.6)	3 (6.0)	8 (16.0)			
Sulfonamides	8 (15.1)	4 (8.0)	8 (16.0)			
Cephalosporins	8 (15.1)	4 (8.0)	2 (4.0)			
	Sex					
Antibiotic Class	Male *n* = 73	Female *n* = 80				
Quinolones	28 (38.4)	37 (46.3)			0.566	0.452
β-lactams	7 (9.6)	9 (11.3)				
Penicillins	11 (15.1)	4 (5.0)				
Aminoglycosides	10 (13.7)	13 (16.3)				
Sulfonamides	10 (13.7)	10 (12.5)				
Cephalosporins	7 (9.6)	7 (8.8)				
	Antibiotics usage					
Antibiotic Class	No *n* = 73	Yes *n* = 80				
Quinolones	28 (38.4)	37 (46.3)			0.668	0.414
β-lactams	7 (9.6)	9 (11.3)				
Penicillins	11 (15.1)	4 (5.0)				
Aminoglycosides	10 (13.7)	13 (16.3)				
Sulfonamides	9 (12.3)	11 (13.8)				
Cephalosporins	8 (11.0)	6 (7.5)				

Numbers in parentheses are percentages.

**Table 8:** Relative frequencies of the bacterial isolates within the cluster dendrogram.

Parameters	Cluster							
	1 *n* = 13	2 *n* = 18	3 *n* = 13	4 *n* = 11	5 *n* = 12	6 *n* = 11	7 *n* = 12	8 *n* = 30
Antibiotics usage								
No	6 (46.2)	7(38.9)	7 (53.9)	10 (90.9)	6 (50.0)	6 (54.6)	3(25.0)	16(53.3)
Yes	7 (53.8)	11 (61.1)	6 (46.1)	1 (9.1)	6 (50.0)	5 (45.4)	9(75.0)	14(46.7)
Genotype								
Local	3 (23.1)	8 (44.4)	6 (46.2)	2(18.2)	4 (33.3)	8 (72.7)	1(8.3)	7(23.3)
FUNAAB Alpha	6 (46.2)	7 (38.9)	0 (0.0)	3(27.3)	3 (25.0)	2 (18.2)	7(58.3)	12(40.0)
Noiler	4 (30.7)	3 (16.7)	7 (53.8)	6(54.5)	5 (41.7)	1 (9.0)	4(33.7)	11(36.7)
Antibiogram (%)								
AMC	84.6	38.8	100	100	100	100	91.7	93.3
CEX	100	100	100	100	100	90.9	75.0	100
CPX	92.3	94.4	100	100	100	100	0.0	100
GEN	100	44.4	100	100	100	100	100	100
PEF	84.6	100	100	100	100	90.9	100	50.0
PEN	100	94.4	76.9	0.0	100	100	100	100
STR	100	100	7.7	100	100	100	100	100
SXT	92.6	100	69.2	81.8	0.0	90.9	100	96.7
NA	100	100	92.3	90.9	100	90.9	100	43.3
OFX	0.0	100	100	100	100	100	100	100
Drug Class								
Quinolones	69.2	98.6	98.1	97.7	100	95.5	75.0	73.3
β-lactams	84.6	38.8	100	100	100	100	91.7	93.3
Penicillins	100	94.4	76.9	0.0	100	100	100	100
Aminoglycosides	100	72.2	53.9	100	100	100	100	71.7
Sulfonamides	92.6	100	69.2	81.8	0.0	90.9	100	100
Cephalosporins	100	100	100	100	100	90.9	75.0	100

Numbers in parentheses are percentages (%); AMC: amoxycillin-clavulanic acid (augmentin); CEX: cephalexin; CPX: ciprofloxacin; CEP: ceporex; GEN: gentamicin; PEF: perfloxacin, PEN: penicillin; SXT: sulfamethoxazole-trimethoprim (co-trimoxazole); STR: streptomycin; NA: nalidixic acid; OFX: ofloxacin.

## Data Availability

The data used to support the findings of this study are available from the corresponding author upon request.

## References

[1] FAOSTAT. https://www.fao.org/faostat/en/#data/QCL.

[2] FAO (2004). Small Scale Poultry Production: A Technical Guide.

[3] FAO CountrySTAT. http://nigeria.countrystat.org/search-and-visualize-data/en/.

[4] Van Boeckel T.P., Brower C., Gilbert M., Grenfell B.T., Levin S.A., Robinson T.P., Teillant A., Laxminarayan R. (2015). Global trends in antimicrobial use in food animals. Proc. Natl. Acad. Sci. USA.

[5] Roth N., Käsbohrer A., Mayrhofer S., Zitz U., Hofacre C., Domig K.J. (2019). The application of antibiotics in broiler production and the resulting antibiotic resistance in *Escherichia coli*: A global overview. Poult. Sci..

[6] Gupta C.L., Blum S.E., Kattusamy K., Druyan D.T., Shapira S., Krifucks R., Zhu O., Yong-Guan Z., Zhou X.-Y., Su J.-Q. (2021). Longitudinal study on the effects of growth-promoting and therapeutic antibiotics on the dynamics of chicken cloacal and litter microbiomes and resistomes. Microbiome.

[7] Zalewska M., Błazejewska A., Czapko A., Popowska M. (2021). Antibiotics and antibiotic resistance genes in animal manure—Consequences of its application in agriculture. Front. Microbiol..

[8] Oluwasile B.B., Agbaje M., Ojo O.E., Dipeolu M.A. (2014). Antibiotic usage pattern in selected poultry farms in Ogun state. Sokoto J. Vet. Sci..

[9] Sanderson H., Fricker C., Brown R.S., Majury A., Liss S.N. (2016). Antibiotic resistance genes as an emerging environmental contaminant. Environ. Rev..

[10] Mehdi Y., Létourneau-Montminy M.-P., Gaucher M.-L., Chorfi Y., Suresh G., Rouissi T., Brar S.T., Cote C., Ramirez A.A., Godbout S. (2018). Use of antibiotics in broiler production: Global impacts and alternatives. Anim. Nutr..

[11] Hedman H.D., Vasco K.A., Zhang L. (2020). A Review of Antimicrobial Resistance in Poultry Farming within Low-Resource Settings. Animals.

[12] Tilman D., Balzer C., Hill J., Befort B.L. (2011). Global food demand and the sustainable intensification of agriculture. Proc. Natl. Acad. Sci. USA.

[13] Hafez H.M., Attia Y.A. (2020). Challenges to the Poultry Industry: Current Perspectives and Strategic Future After the COVID-19 Outbreak. Front. Vet. Sci..

[14] Selaledi A.L., Mohammed H.Z., Manyelo T.G., Mabelebele M. (2020). The Current Status of the Alternative Use to Antibiotics in Poultry Production: An African Perspective. Antibiotics.

[15] Yang Y., Ashworth A.J., Willett C., Cook K., Upadhyay A., Owens P.R., Ricke S.C., DeBruyn J.M., Moore P.A. (2019). Review of Antibiotic Resistance, Ecology, Dissemination, and Mitigation in U.S. Broiler Poultry Systems. Front. Microbiol..

[16] UK-VARSS Veterinary Antibiotic Resistance and Sales Surveillance Report (UK-VARSS 2019). www.gov.uk/government/collections/veterinary-antimicrobialresistance-and-sales-surveillance.

[17] Bean-Hodgins L., Kiarie E.G. (2021). Mandated restrictions on the use of medically important antibiotics in broiler chicken production in Canada: Implications, emerging challenges, and opportunities for bolstering gastrointestinal function and health—A review. Can. J. Anim. Sci..

[18] Bushen A., Tekalign E., Abayneh M. (2021). Drug- and Multidrug-Resistance Pattern of Enterobacteriaceae Isolated from Droppings of Healthy Chickens on a Poultry Farm in Southwest Ethiopia. Infect. Drug Resist..

[19] Mkize N., Zishiri O.T., Mukaratirwa S. (2017). Genetic characterisation of antimicrobial resistance and virulence genes in *Staphylococcus aureus* isolated from commercial broiler chickens in the Durban metropolitan area, South Africa. J. S. Afr. Vet. Assoc..

[20] Ahlers C., Alders R.G., Bagnol B., Cambaza A.B., Harun M., Mgomezulu R., Msami H., Pym B., Wegener P., Wethli E. (2009). Improving Village Chicken Production: A Manual for Field Workers and Trainers.

[21] Hegde N.V., Kariyawasam S., DebRoy C. (2016). Comparison of antimicrobial resistant genes in chicken gut microbiome grown on organic and conventional diet. Vet. Anim. Sci..

[22] Guèye E.F. (1999). Ethnoveterinary medicine against poultry diseases in African villages. World’s Poult. Sci. J..

[23] Abo-EL-Sooud K. (2018). Ethnoveterinary perspectives and promising future. Int. J. Vet. Sci. Med..

[24] Yakubu A., Bamidele O., Hassan W.A., Ajayi F.O., Ogundu U.E., Alabi O., Sonaiya E.B., Adebambo O.A. (2020). Farmers’ choice of genotypes and trait preferences in tropically-adapted chickens in five agro-ecological zones in Nigeria. Trop. Anim. Health Prod..

[25] Bamidele O., Sonaiya E.B., Adebambo O.A., Dessie T. (2020). On-station performance evaluation of improved tropically adapted chicken breeds for smallholder poultry production systems in Nigeria. Trop. Anim. Health Prod..

[26] Bamidele O., Amole T.A., Oyewale O.A., Bamidele O.O., Yakubu A., Ogundu U.E., Ajayi F.O., Hassan W.A. (2022). Antimicrobial Usage in Smallholder Poultry Production in Nigeria. Vet. Med. Int..

[27] Alabi O.O., Ajayi F.O., Bamidele O., Yakubu A., Ogundu E.U., Sonaiya E.B., Ojo M.A., Hassan W.A., Adebambo O.A. (2020). Impact assessment of improved chicken genetics on livelihoods and food security of smallholder poultry farmers in Nigeria. Livest. Res. Rural. Dev..

[28] Hoffmann I. (2010). Livestock biodiversity. Rev. Sci. Tech..

[29] Kryger K.N., Thomsen K.A., Whyte M.A., Dissing M., FAO (2010). Smallholder Poultry Production—Livelihoods, Food Security and Sociocultural Significance.

[30] VKM (2016). Antimicrobial Resistance Due to the Use of Biocides and Heavy Metals: A Literature Review.

[31] Westphal-Settele K., Konradi S., Balzer F., Schönfeld J., Schmithausen R. (2018). Die Umwelt als Reservoir für Antibiotikaresistenzen: Ein wachsendes Problem für die öffentliche Gesundheit? [The environment as a reservoir for antimicrobial resistance: A growing problem for public health?]. Bundesgesundheitsblatt Gesundh. Gesundh..

[32] Robles-Jimenez L.E., Aranda-Aguirre E., Castelan-Ortega O.A., Shettino-Bermudez B.S., Ortiz-Salinas R., Miranda M., Li X. (2021). Worldwide Traceability of Antibiotic Residues from Livestock in Wastewater and Soil: A Systematic Review. Animals.

[33] Teale C.J. (2002). Antimicrobial resistance and the food chain. J. Appl. Microbiol. Symp. Suppl..

[34] FAO (2015). The Economic Lives of Smallholder Farmers: An Analysis Based on Household Data from Nine Countries.

[35] Swift B.M.C., Bennett M., Waller K., Dodd C., Murray A., Gomes R.L., Humphreys B., Hobman J.L., Jones M.A., Whitlock S.E. (2019). Anthropogenic environmental drivers of antimicrobial resistance in wildlife. Sci. Total Environ..

[36] Franklin A.M., Williams C.F., Andrews D.M., Woodward E.W., Watson J.E. (2016). Uptake of three antibiotics and an antiepileptic drug by wheat crops spray irrigated with wastewater treatment plant effluent. J. Environ. Qual..

[37] Franklin A.M., Williams C.F., Watson J.E. (2018). Assessment of Soil to Mitigate Antibiotics in the Environment Due to Release of Wastewater Treatment Plant Effluent. J. Environ. Qual..

[38] Tripathi V., Cytryn E. (2017). Impact of anthropogenic activities on the dissemination of antibiotic resistance across ecological boundaries. Essays Biochem..

[39] Fekede G., Tadesse Y., Esatu W., Dessie T. (2021). On-farm comparative production and reproduction performance evaluation of Sasso, Sasso-RIR, Koekoek and Improved Local chicken breeds in Bako Tibe and Dano districts of western Oromia, Ethiopia. Livest. Res. Rural. Dev..

[40] Guni F.S., Mbaga S.H., Katule A.M. (2021). Performance Evaluation of Kuroiler and Sasso Chicken Breeds Reared under On-farm and On-station Management Conditions in Tanzania. Eur. J. Agric. Food Sci..

[41] Awogbemi J., Adeyeye M., Akinkunmi E.O. (2018). A Survey of Antimicrobial Agents Usage in Poultry Farms and Antibiotic Resistance in *Escherichia coli* and Staphylococci Isolates from the Poultry in Ile-Ife, Nigeria. J. Infect. Dis. Epidemiol..

[42] Sule I.O., Olorunfemi A.A., Otori A.O. (2019). Mycological and bacteriological assessment of poultry droppings from poultry pens within Ilroin, Kwara, Nigeria. Sci. World J..

[43] King D.E., Malone R., Lilley S.H. (2000). New classification and update on the quinolone antibiotics. Am. Fam. Physician..

[44] Osman A.Y., Elmi S.A., Simons D., Elton L., Haider N., Khan M.A., Othman I., Zumla A., McCoy D., Kock R. (2021). Antimicrobial Resistance Patterns and Risk Factors Associated with *Salmonella* spp. Isolates from Poultry Farms in the East Coast of Peninsular Malaysia: A Cross-Sectional Study. Pathogens.

[45] Ngogang M.P., Ernest T., Kariuki J., Mouliom Mouiche M.M., Ngogang J., Wade A., van der Sande M.A.B. (2021). Microbial Contamination of Chicken Litter Manure and Antimicrobial Resistance Threat in an Urban Area Setting in Cameroon. Antibiotics.

[46] Kakooza S., Muwonge A., Nabatta E., Eneku W., Ndoboli D., Wampande E., Munyiirwa D., Kayaga E., Tumwebaze M.A., Afayoa M. (2021). Retrospective analysis of antimicrobial resistance in pathogenic *Escherichia coli* and *Salmonella* spp. isolates from poultry in Uganda. Int. J. Vet. Sci. Med..

[47] WHO Antimicrobial Resistance. https://www.who.int/news-room/fact-sheets/detail/antimicrobial-resistance.

[48] Martínez J.L. (2017). Effect of antibiotics on bacterial populations: A multi-hierachical selection process. F1000Research.

[49] Trawińska B., Chmielowiec-Korzeniowska A., Nowakowicz-Dębek B., Tymczyna L., Bombik T., Pyrz M., Tymczyna-Sobotka M. (2016). Evaluation of microbial contamination of feces and soil on a laying-hen farm depending on sampling site and season. Rev. Bras. Zootec..

[50] do Amaral L.A. (2004). Drinking Water as a Risk Factor to Poultry Health. Braz. J. Poult. Sci..

[51] Rose N., Besuderau F., Drouin P., Toux J.Y., Rose V., Colin P. (2000). Risk factors of Salmonella persistence after cleansing and disinfection in French broiler—Chicken house. Prevent. Vet. Med..

[52] Kolář M., Pantůček R., Bardoň J., Vágnerová I., Typovská H., Válka I., Doškař J. (2002). Occurrence of antibiotic-resistant bacterial strains isolated in poultry. Vet. Med. Czech..

[53] Ajayi K.O., Omoya F.O. (2017). Antibiotic Usage Pattern in Poultry and Resistance Pattern of Human Pathogenic Bacteria Isolated from Poultry Droppings in Akure, Nigeria. Int. J. Biomed. Sci. Eng..

[54] Nhung N.T., Chansiripornchai N., Carrique-Mas J.J. (2017). Antimicrobial Resistance in Bacterial Poultry Pathogens: A Review. Front. Vet. Sci..

[55] Xu J., Sangthong R., McNeil E., Tang R., Chongsuvivatwong V. (2020). Antibiotic use in chicken farms in northwestern China. Antimicrob. Resist. Infect. Control.

[56] Liu C., Wang P., Dai Y., Liu Y., Song Y., Yu L., Feng C., Liu M., Xie Z., Shang Y. (2021). Longitudinal monitoring of multidrug resistance in *Escherichia coli* on broiler chicken fattening farms in Shandong, China. Poult. Sci..

[57] Afunwa R.A., Ezeanyinka J., Afunwa E.C., Udeh A.S., Oli N.A., Unachukwu M. (2020). Multiple antibiotic resistant index of gram-negative bacteria from bird droppings in two commercial poultries in Enugu, Nigeria. Open J. Med. Microbiol..

[58] Mpenda F.N., Schilling M.A., Campbell Z., Mngumi E.B., Buza J. (2019). The genetic diversity of local african chickens: A potential for selection of chickens resistant to viral infections. J. Appl. Poult. Res..

[59] Rotchell D., Paul D. (2016). Multiple Antibiotic Resistance Index. Fitness and Virulence Potential in Respiratory *Pseudomonas aeruginosa* from Jamaica. J. Med. Microbiol..

[60] Gheyas A.A., Vallejo-Trujillo A., Kebede A., Lozano-Jaramillo M., Dessie T., Smith J., Hanotte O. (2021). Integrated Environmental and Genomic Analysis Reveals the Drivers of Local Adaptation in African Indigenous Chickens. Mol. Biol. Evol..

[61] Riaz S., Faisal M., Hasnain S. (2011). Antibiotic Susceptibility Pattern and Multiple Antibiotic Resistance (MAR) Calculation of Extended Spectrum β-Lactamase (ESBL) Producing *Escherichia coli* and Klebsiella Species in Pakistan. Afr. J. Biotechnol..

[62] WHO (2017). Global Priority List of Antibiotic-Resistant Bacteria to Guide Research, Discovery and Development of New Antibiotics. https://www.who.int/medicines/publications/WHO-PPL-Short_Summary_25Feb-ET_NM_WHO.pdf.

[63] WHO (2021). The Selection and Use of Essential Medicines: Report of the WHO Expert Committee on Selection and Use of Essential Medicines.

[64] Murray C.J.L., Ikuta K.S., Sharara F., Swetschinski L., Aguilar G.R. (2022). Global burden of bacterial antimicrobial resistance in 2019: A systematic analysis. Lancet.

[65] Aworh M.K., Kwaga J.K.P., Hendriksen R.S., Okolocha E.C., Thakur S. (2021). Genetic relatedness of multidrug resistant *Escherichia coli* isolated from humans, chickens and poultry environments. Antimicrob. Resist. Infect. Control.

[66] Wang Y., Lyu N., Liu F., Liu W.J., Bi Y., Zhang Z., Ma S., Cao J., Song X., Wang A. (2021). More diversified antibiotic resistance genes in chickens and workers of the live poultry markets. Environ. Int..

[67] Hull D.M., Harrell E., van Vliet A.H.M., Correa M., Thakur S. (2021). Antimicrobial resistance and interspecies gene transfer in *Campylobacter coli* and *Campylobacter jejuni* isolated from food animals, poultry processing, and retail meat in North Carolina, 2018–2019. PLoS ONE.

[68] Suwono B., Eckmanns T., Kaspar H., Merle R., Zacher B., Kollas C. (2021). Cluster analysis of resistance combinations in *Escherichia coli* from different human and animal populations in Germany 2014–2017. PLoS ONE.

[69] Alhaji N.B., Isola T.O. (2018). Antimicrobial usage by pastoralists in food animals in North-central Nigeria: The associated socio-cultural drivers for antimicrobials misuse and public health implications. One Health.

[70] Bamidele O., Amole T.A. (2021). Impact of COVID-19 on Smallholder Poultry Farmers in Nigeria. Sustainability.

[71] Cheesbrough M. (2006). District Laboratory Practice in Tropical Countries.

[72] Ochei J., Kolhatkar A. (2008). Medical Laboratory Science: Theory and Practice.

[73] WHO AWaRe Classification. https://www.who.int/publications/i/item/2021-aware-classification.

[74] Krumpermann P.H. (1983). Multiple antibiotic resistance indexing of *Escherihia coli* to identify high-risk sources of fecal contamination in foods. Appl. Environ. Microbiol..

[75] Nattino G., Pennell M.L., Lemeshow S. (2020). Assessing the goodness of fit of logistic regression models in large samples: A modification of the Hosmer-Lemeshow test. Biometrics.

[76] IBM SPSS (2020). Statistics for Windows, Version 27.0.

[77] R Core Team (2018). R: A Language and Environment for Statistical Computing.

[78] Kassambara A., Mundt F. (2020). Factoextra: Extract and Visualize the Results of Multivariate Data Analyses, R Package Version 1.0.7. https://CRAN.R-project.org/package=factoextra.

